# Magnetically controlled planar hyperbolic metamaterials for subwavelength resolution

**DOI:** 10.1038/srep18172

**Published:** 2015-12-11

**Authors:** Bo Han Cheng, Hong Wen Chen, Kai Jiun Chang, Yung-Chiang Lan, Din Ping Tsai

**Affiliations:** 1 Research Center for Applied Sciences, Academia Sinica, Taipei 115, Taiwan; 2 Department of Photonics and Advanced Optoelectronic Technology Center, National Cheng Kung University, Tainan 70101, Taiwan; 3 Department of Physics, National Taiwan University, Taipei 10617, Taiwan

## Abstract

Breaking diffraction limitation is one of the most important issues and still remains to be solved for the demand of high-density optoelectronic components, especially for the photolithography industry. Since the scattered signals of fine feature (i.e. the size is smaller than half of the illuminating wavelength *λ*) are evanescent, these signals cannot be captured by using conventional glass- or plastic-based optical lens. Hence the corresponding fine feature is lost. In this work, we propose and analyze a magnetically controlled InSb-dielectric multi-layered structure with ability of subwavelength resolution at THz region. This layered structure can resolve subwavelength structures at different frequencies merely changing the magnitude of external magnetic field. Furthermore, the resolving power for a fixed incident frequency can be increased by only increasing the magnitude of applied external magnetic field. By using transfer matrix method and effective medium approach, the mechanism of achieving super resolution is elucidated. The electromagnetic numerical simulation results also prove the rationality and feasibility of the proposed design. Because the proposed device can be dynamically reconfigured by simply changing the magnitude of external magnetic field, it would provide a practical route for multi-functional material, real-time super-resolution imaging, and photolithography.

Recently, for acquiring subwavelength image, some plasmonic-based artificial materials that possess the ability to deliver high spatial frequencies (which correspond to fine features of the objects to be resolved) have been explored, such as superlens^1^,^2^,^3^, multi-layered based hyperlens^4^,^5^,^6^,^7^,^8^,^9^,^10^ and hybrid-superlens hyperlens^11^,^12^,^13^. However, the constituent conditions of the used materials that are required to satisfy the criteria of achieving subwavelength resolution are quite rigorous. Hence the working wavelength band region is very limited (i.e. single-wavelength resolution) for the highly dispersive characteristic of the materials^4^,^11^. From a practical point of view, broadening the bandwidth of achieving subwavelength resolution is an important issue for many application-related imaging techniques.

The indium antimonide (InSb)–dielectric interface can support propagation of surface plasmon (SPs) under illumination of terahertz light source^14^. In an external magnetic field, the SPs are transformed into the surface magnetoplasmons (SMPs)^15^. Due to the tunable optical properties of InSb (which can be tailored by external magnetic field^16^, temperature^17^, and doping^18^), it has attracted much attention from the entire world. Recently, many InSb-based tunable optoelectronic components (they are managed by applying an external magnetic field) have been investigated, such as tunable terahertz metamaterial^19^, broadly tunable one-way waveguide^20^, actively focal-length-control plane lens^21^ and magnetic lens^22^. The coupling effects between the constituent materials via SMPs that exist in the above mentioned function also play an important role in the layered system with the function of subwavelegth solution^4^,^23^,^24^. Therefore, by replacing the metal material with InSb, a periodically arranged InSb-dielectric multi-layered hyperlens at the THz region with adjustable resolving power and operating frequency will be achieved.

In order to obtain subwavelength imaging with the inclusion of tunability and realizability, the propagation behavior of the scattered signals from the objects to be resolved (especially the information of high order spatial frequency) in the hyperlens should be well controlled. Furthermore, the energy of corresponding information cannot be dissipated until it is captured by the detector (or microscopy). Here, we use a multi-layered InSb-dielectric structure under an external magnetic field as the hyperlens in which all the scattered signals can be transferred from the source to the detector. In order to accomplish the desired hyperlens, the optical properties (effective relative permittivity) and isofrequency curve of InSb-dielectric multi-layered structure under various magnitude of external magnetic field are investigated first. The corresponding requirements of achieving subwavelength resolution under specific values of external magnetic are also derived. Finally, the abilities of subwavelength resolution of the proposed structures with adjustable resolving power and operating frequency are demonstrated. We point out a practical approach to control all the diffraction signals toward a specific direction by using transfer matrix method and effective medium theory. Numerical calculations and computer simulation (based on finite element method, FEM) are carried out, which agree well with each other.

## Results

**Figure 1:**
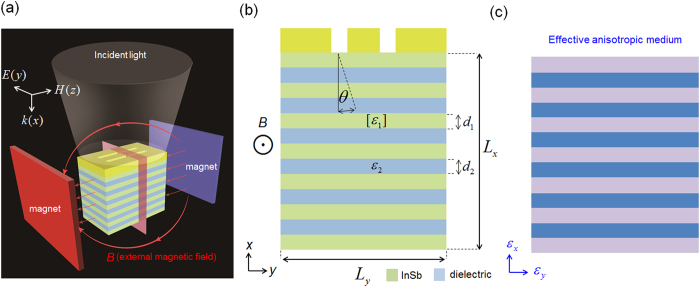
Concept for magnetically controlled subwavlength resolution setup. (**a**) A diagram of setup. (**b**) Side-view of the layered semiconductor-dielectric structure under an external magnetic field *B* (viewed from the gray cut plane shown in (**a**)). The center-to-center distance between two resolved slits carved on Cr mask is 100 *μ*m. The width of the both resolved slits is 50 *μ*m. *L*_x_ = 130 *μ*m (*L*_y_ = 440 *μ*m) is the *x*-direction (*y-*direction) length of layered system. *d*_1_ = *d*_2_ = 10 *μ*m. *θ* denotes the angle between the propagation direction and the normal direction. (**c**) Structure equivalent to (**b**), which includes the effect of external magnetic field.

Figure 1(a) presents the schematic diagram of investigated structure under the illumination of THz light source. The semiconductor and dielectric materials are InSb and PMMA, respectively (Method). The dielectric constant of PMMA is set to 2.5. A chromium (Cr) mask (with two slits, which represent the resolved objects) is put on the top of the periodically arranged multi-layered structure. A uniform magnetic field *B* is applied in the system, which can be accomplished by using two large magnets with different magnetism. Here, the Voigt configuration (*B* is parallel to the interface between different media and perpendicular to the wave propagation direction *k*^15^) and TM mode incident light source (the electric fields are in the *xy*-plane) are considered. In this configuration, the components of dielectric tensor of the semiconductor depend on the magnitude of *B*. (tensor, Method)

To prove our concept, the propagation property of delivered wave (after passing through the slits, as shown in Fig. 1(b)) in the layered system should be clarified first. Considering the Maxwell–Faraday equation 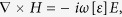 the *H*_*z*_ and *E*_*y*_ components in the semiconductor (the subscript 1) and dielectric (the subscript 2) regions can be respectively expressed as,


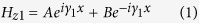



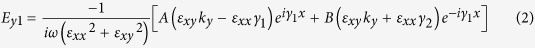



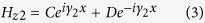



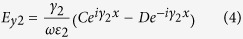


where 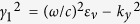 and 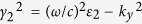; 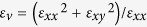 is Voigt dielectric constant of InSb. Further, taking into account the continuous boundary and Bloch conditions at the InSb-dielectric interface (that is, 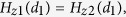 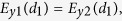

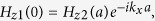
 and 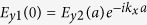, where *a* = *d*_1_ + *d*_2_), the nontrivial solution would be obtained by calculating the determine shown as below,


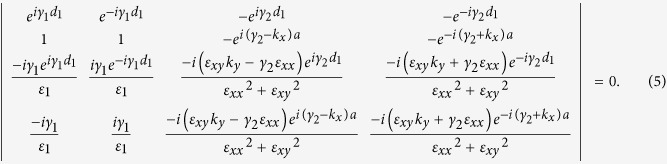


Reorganizing Eq. (5) will yield the dispersion relation of the layered structure (see Appendix A of supplementary information)


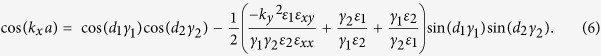


Equation (6) can be further simplified by using Taylor expansion (up to second order), a long wavelength approximation 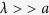 (*λ* and 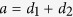 denote the incident wavelength in free space and period length, respectively), and considering 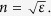 The dispersion relation of the layered system with inclusion of the effect of external magnetic field will become as bellow


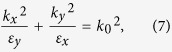


where 
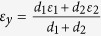
 and 
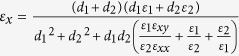
 are the effective relative permittivities of the equivalent anisotropic material in the *y* and *x*-directions, respectively, as shown in Fig. 1(c); *k*_0_ is the wave vector in free space.

**Figure 2:**
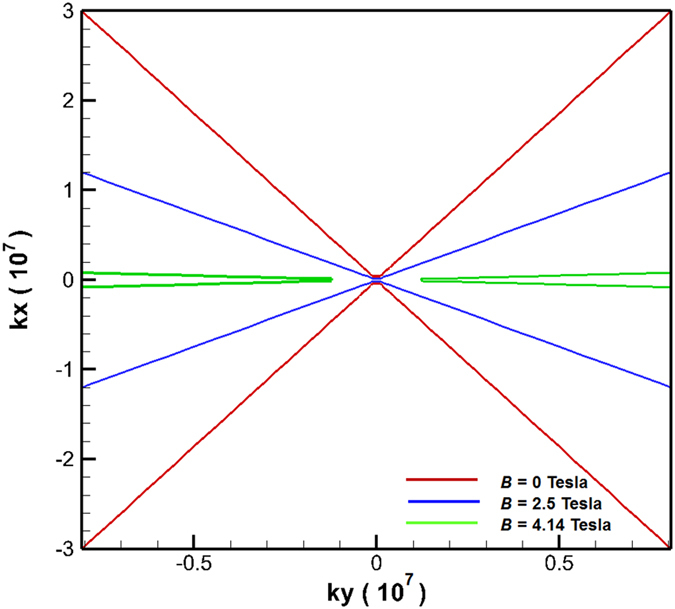
Isofrequency curves in wavevector space for light propagating in the layered structure under various values of external magnetic *B* at 1 THz. Blue (green, red) line shows an hyperbolic type of dispersion relation that corresponds to *B* = 0 Tesla (2.5 Tesla, 4.14 Tesla). The real parts of (*ε*_*x*_, *ε*_*y*_) for blue, green and red lines are (5.088, −70.34), (5.51, −12.21) and (0.0545, −0.2734), respectively.

**Figure 3:**
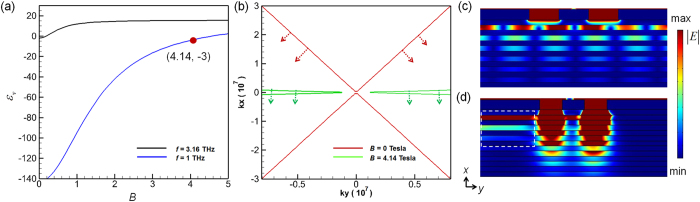
Magnetically controlled *ε*_*v*_ values and isofrequency curves in a layered semiconductor-dielectric structure. (**a**) Calculated *ε*_*v*_ values as a function of magnitude of external magnetic field *B* at 1 THz and 3.16 THz. (**b**) Calculated isofrequency curves at 1 THz with magnitudes of external magnetic field of 0 Tesla and 4.14 Tesla. (**c**,**d**) Simulated time-averaged power flow contours with magnitudes of external magnetic field equal to 0 Tesla and 4.14 Tesla, respectively.

For achieving subwavelength resolution, the isofrequency dispersion curves of the proposed layered structure need to be hyperbolic form and as flat as possible. To meet this requirement, the conditions: 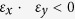 and 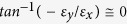 need to be satisfied^4^,^5^. (As 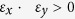 occurs, the dispersion curve would be elliptic. See Appendix B of supplementary information.) Under an external magnetic field, the conditions are transferred to 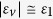 (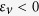) and 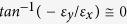. (Method) (Note that, in this study, the dielectric constant of PMMA is set to 2.5. For fulfilling the above mentioned criteria, the value of *ε*_*v*_ should be close to −2.5.) Moreover, since the effective dielectric tensor of InSb is altered by the applied external magnetic field, the isofrequency dispersion curve is also expected to change with the external magnetic field. Figure 2 plots the isofrequency dispersion curves (based on numerical calculations of Eq. (7)) of the investigated layered structure under various values of external magnetic field *B* at incident frequency of 1 THz. Figure 2 shows that, when no magnetic field is applied, the designed layered structure in Fig. 1(b) possesses the hyperbolic isofrequency dispersion curve. However, the slope (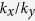) of the hyperbolic curve is large. Figure 2 also indicates that, when the magnetic field is applied, the dispersion curves still keep the hyperbolic form. However, the slope of the curve decreases with the increase of the magnitude of magnetic field. It implies that the wave’s propagation properties can be manipulated by the applied magnetic field. It is worth to mention that, for the goal of subwavelength resolution, the operating condition of red line (*B* = 4.14 Tesla) in Fig. 2 is better because of its ultra-flat hyperbolic curve^4^,^5^. All the scattered signals come from the objects to be resolved (the two slits) in the layered system can transfer to the output side without producing interference. The corresponding simulation result for achieving subwavelength resolution is shown in Fig. 3(d).

Figure 3(a) plots the calculated values of *ε*_*v*_ versus the magnitude of external magnetic field *B* at incident light frequencies (*f*  ) of 1 THz and 3.16 THz. For incident frequency of 1 THz, Fig. 3(a) indicates that the value of *ε*_*v*_ is −3.0 as the magnitude of applied magnitude *B* equals to 4.14 Tesla. It implies that the two slits can be successfully resolved by using this value of *B* (around 4.14 Tesla) at 1 THz. Using other values of *B*, the subwavelength resolution would not be achieved due to violation of 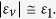 Conversely, for *f* = 3.16 THz (also in Fig. 3(a)), no suitable value of *B* can achieve subwavelength resolution because all the values of *ε*_*v*_ are positive. Figure 3(b) redraws the isofrequency curves at 1 THz (Fig. 2) for *B* equal to 0 and 4.14 Tesla. The same hyperbolic form but different slope reveals that, for both *B* values, all the excited high-order evanescent signals can be transferred into propagation mode inside the layered structure but have different propagation directions. The propagation direction in the layered structure can be determined by the formula 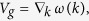 as the arrows shown in Fig. 3(b). For the case of *B* = 4.14 Tesla, the slope of dispersion curve is very small and the pass-through signals will propagate along *x*-direction. Hence, the interference between the scattered waves from the two slits is expected to be avoidable. The corresponding simulated results for *B* equal to 0 and 4.14 Tesla at 1 THz are conducted and presented in Fig. 3(c,d), respectively. Figure 3(d) displays that the two slits can be resolved at the end of the proposed structure for *B* = 4.14 Tesla, which is consistent with analyses of the dispersion curves. Notably, Fig. 3(d) also exhibits a unidirectional SMPs propagation along –y direction. The propagation of SMPs along + *y* direction is suppressed (marked by the white dashed line in Fig. 3(d)). Furthermore, it is non-reciprocal (i.e. the propagation direction of SMPs will be opposite when the direction of the applied magnetic field is reversed). The non-symmetrical and non-reciprocal properties of SMP are ascribed to the combined effect of cyclotron motion and plasma oscillation of free electron in the semiconductor^25^ (See Appendix C of supplementary information).

**Figure 4:**
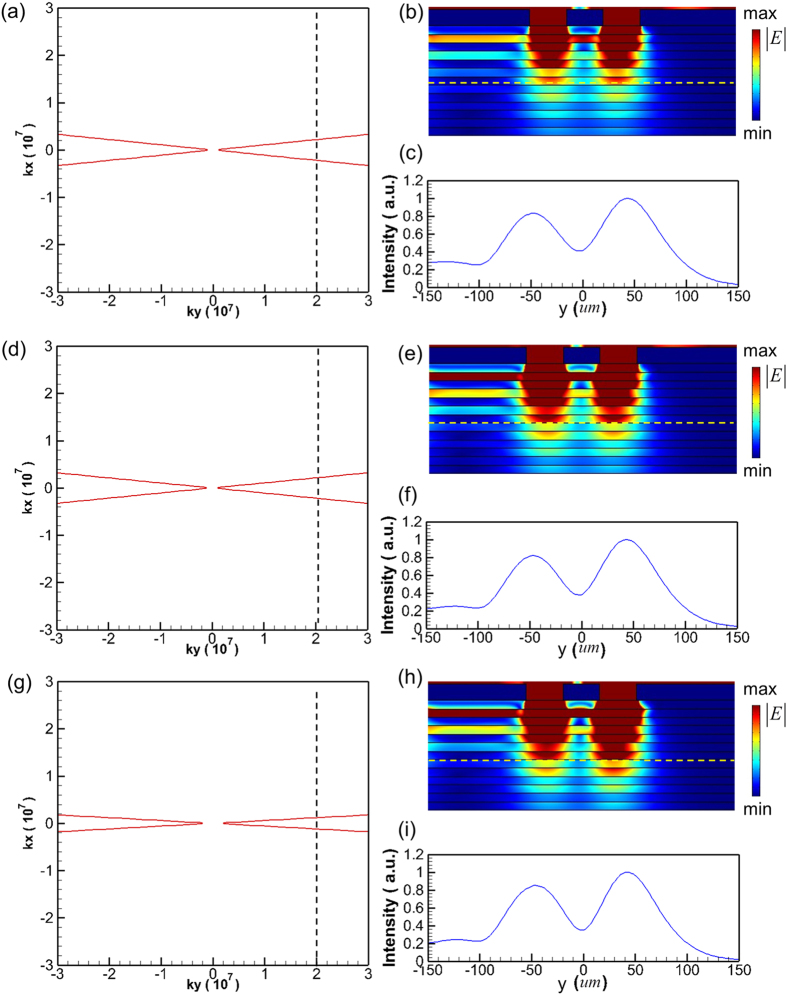
Calculated isofrequency curves and simulated time-averaged power flow contours. (**a**,**d**,**g**) Isofrequency curves with parameters (*f, B*) equal to (1.1 THz, *B* = 3.66 Tesla), (1.2 THz, *B* = 3.26 Tesla), and (1.3 THz, *B* = 2.94 Tesla), respectively. (**b**,**e**,**h**) Simulated time-averaged power flow contours at operating conditions of (**a**,**d**,**g**) respectively. (**c**,**f**,**i**) Normalized intensity versus y position measured at the dashed yellow lines in (**b**,**e**,**h**) respectively.

As mentioned above, the value of *ε*_*v*_ can be tuned by changing the magnitude of external magnetic field *B*. Our calculations reveal that, for a fixed center-to-center distance of the slits (here 100 *μ*m), the resolvable incident frequency gradually increases as reducing the value of *B* within some specific frequency region. For instance, the magnetic field decreases from 3.66 to 2.94 Tesla, the corresponding resolved frequency increases from 1.1 to 1.3 THz. To verify this prediction, the corresponding simulation results are also provided. Figure 4(a,d,g) present the calculated isofrequencies for the structure of Fig. 1(b) under the values of applied magnetic field and incident frequency, (*B, f*  ), equal to (3.66 Tesla, 1.1 THz), (3.26 Tesla, 1.2 THz), and (2.94 Tesla, 1.3 THz), respectively. In Fig. 4(a,d,g), the maximum value of transverse wavevector (k_y_) of the separated slits is denoted as the black dashed line. Here this value is defined as the first zero of sinc function (i.e. the Fourier transformation of a rectangular function). Following the formula 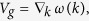 the corresponding propagation directions are nearly parallel to the *x*-axis. Therefore, the two slits can be successfully resolved on the other side of the hyperlens as shown in Fig. 4(b,e,h).

**Figure 5:**
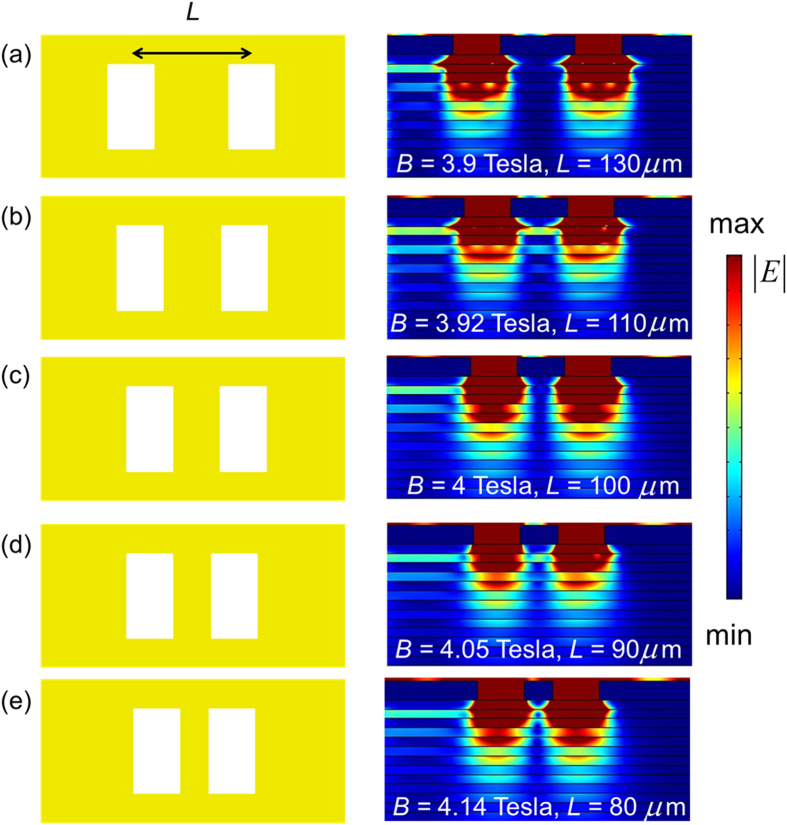
Simulated minimum resolvable distance between two slits for different values of applied magnetic field at incident frequency of 1 THz. The center-to-center distance (*L*) of resolved slits and magnitude of applied magnetic field (*B*) are (**a**) *L* = 130 *μ*m and *B* = 3.9 Tesla, (**b**) *L* = 110 *μ*m and *B* = 3.92 Tesla, (**c**) *L* = 100 *μ*m and *B* = 4 Tesla, (**d**) *L* = 90 *μ*m and *B* = 4.05 Tesla, and (**e**) *L* = 80 *μ*m and *B* = 4.14 Tesla.

Finally, the dependence of achievable resolving power on the magnitude of external magnetic field for a fixed incident frequency is examined. Figure 5 plots the simulation results of the minimum resolvable distance between two slits for different values of magnetic field at 1 THz. Figure 5 indicates that, the resolving power of the layered structure is increased by only increasing the magnitude of the applied magnetic field (the incident frequency and structure are unchanged). Therefore, in the proposed layered structure, the resolving power can be manipulated by the magnitude of applied magnetic field that is similar to the adjustable object lenses of a microscope with different magnifications. Some other useful phenomena based on the SMPs, such as magneto-optical transparency^26^ and interference-induced transparency^27^, can also be implemented by using the proposed design. Moreover, due to its tunability (including the effective permittivity and the value of *B*), the proposed layered structure has a wide range of potential application in different field such as magnetic recording^28^, magnetoplasmonic crystal^29^, and one-way waveguide^30^.

**Figure 6:**
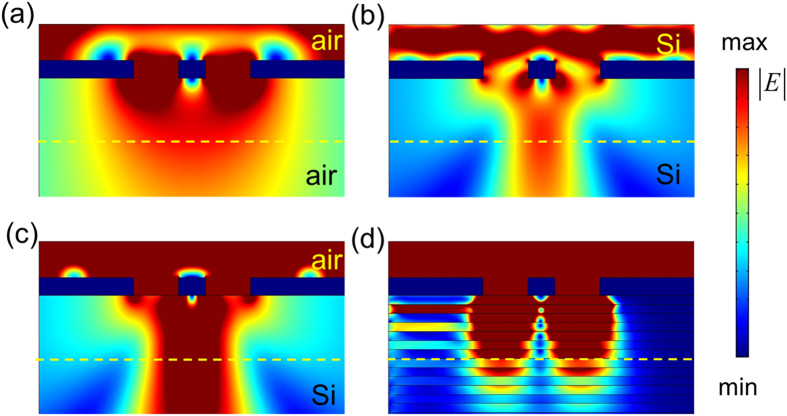
Simulated time-averaged power flow contours for double slits embedded in different materials at incident frequency of 1 THz. (**a**,**b**) The double slits are embedded in air and in Si, respectively. (**c**,**d**) The imaging device under the double slits are Si plate and proposed layered structure with *B* = 4.14 Tesla, respectively. *L* = 80 *μ*m.

For further demonstrating the ability of subwavelength resolution of the proposed structures, the images of double slits embedded in various materials are also examined. Figure 6(a,b) plot the simulated time-averaged power flow contours for the double slits embedded in air and in Si (Method), respectively, at 1 THz. In addition, Fig. 6(c) presents the same simulated contours for the double slits on a uniform Si plate. For comparison, the simulated contours in Fig. 5(e) are replotted in Fig. 6(d). The corresponding normalized intensities versus y position measured at the dashed yellow lines (the measure position is the same as Fig. 4) are also plotted in Supplementary Information (Appendix D). Figure 6(a–c) indicate that the two slits can be distinguished at the x position very close to the slits. However, at the position of dashed yellow line, the double slits only can be resolved by the proposed layered structure with *B* = 4.14 Tesla.

Notably, the loss in the proposed layered structure cannot be ignored. The decay of intensity of the propagating wave in the multilayered structure is also examined (See Appendix E of Supplementary Information). The simulation result shows that the intensity of the propagating wave at the position of image measurement (i.e. the dashed yellow lines in Fig. 6) is reduced to about one-twentieth of that at the exit of slit. However, as demonstrated in Fig. 6, the double slits only can be resolved by the proposed layered structure at this position and at the bottom of the structure. Actually, if we add more layers in the layered structure, the double slits are still resolved at the bottom of the structure. Therefore, our proposed concept for subwavelength resolution is useful.

## Discussion

A semiconductor-based hyperlens with magnetic tunability at terahertz region is proposed and analyzed. The FEM electromagnetic simulations verify the design of the proposed structure to achieve subwavelength resolution. The super resolution of the proposed device under different frequencies is accomplished by merely changing the magnitude of the external magnetic field. The resolving power for a fixed incident frequency can be increased by only increasing the magnitude of applied magnetic field. More importantly, to illustrate the physical reliability, the constituent materials of achieving super resolution are available in nature. Hence, our methodology provide a way to multi-functional material, real-time subwavelength imaging, and high-density optoelectronic components with considering the effects of abnormal diffraction feature. It will provide us a path to overcome the major issue of biological image studies.

## Methods

All the simulated results shown in Figs 3, 4, 5 are conducted from the commercial electromagnetic software COMSOL Multiphysics based on the finite element numerical method. For modeling our proposed subwavelength resolution, a layered structure and two resolved slits (the center-to-center distance is smaller than half of *λ*) carved on Cr mask are investigated, as shown in Fig. 1(b). Here, a practical design based on existing materials and experiment technique is presented to illustrate that the function of subwavelength resolution can be really achieved. The simulated dielectric tensor of InSb with considering the Voigt configuration can be written as^15^,


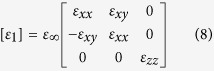


where 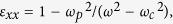 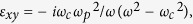 
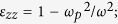
 *ω* is the angular frequency of incident wave; and 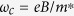 presents the cyclotron frequency, which can be tuned by the applied magnetic field. 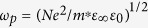 is the plasma frequency, where N, e, m^*^ are the density, the electron charge, and the effective mass of free electron, respectively. *ε*_∞_(*ε*_0_) is the high-frequency (vacuum) permittivity. Note that, the used parameters of InSb at room temperature are *ω*_*p*_ = 2 × 10^13^(rad/s), *ε*_∞_ = 15.68, and m^*^ = 0.014*m*^0^ 31. The investigated values of magnetic field *B* are also within an available range^32^. The dielectric constant at investigated region of PMMA is 2.5, which is adopted from literature^33^. The dielectric constant of Si at terahertz region comes from literature^34^ (which is about 3.4 at 1 THz). A TM-polarized light (polarized in the y direction) is normally incident from the top. The perfectly matched layers (PMLs) are used in all propagating directions.

Note that, the both two cases shown in Fig. 3(b) fulfill the basic requirement (i.e. the hyperbolic dispersion relation) that the evanescent higher-order modes are transferred into propagating modes inside the multi-layered structure. For resolving subwavelength objects, the propagation direction of the corresponding energy also needs to be determined. Due to 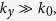 the dispersion relation Eq. (7) can be simplified as


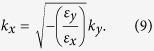


The propagation direction of the transferred signals can be decided by


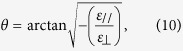


where *θ* presents the angle between the propagation direction and the normal direction (see Fig. 1(b)) inside the layered structures. For obtaining higher resolution, *θ* needs to be as small as possible. A smaller value of *θ* will prevent the interference between the tunneling signals that come from the two slits. Conventionally, by fine tuning the parameters *d*_1_, *d*_2_, *ε*_1_ and *ε*_2_, both the effective parallel permittivity *ε*_*y*_ nearly equal to zero and a small value of *θ* can be obtained. Here we demonstrate that only tuning the magnitude of applied magnetic field *B* can also achieve this purpose in the proposed layered structures. The simulated time-averaged power flow contours are shown in Fig. 3(c,d).

## Additional Information

**How to cite this article**: Cheng, B. H. *et al.* Magnetically controlled planar hyperbolic metamaterials for subwavelength resolution. *Sci. Rep.* **5**, 18172; doi: 10.1038/srep18172 (2015).

## Supplementary Material

Supplementary Information
