# Supplementary material for: Magnetically controlled planar hyperbolic metamaterials for subwavelength resolution
Source: Sci Rep. 2015 Dec 11;5:18172. doi: 10.1038/srep18172 (PMC4676063; doi:10.1038/srep18172)
Supplement: Supplementary Information [file srep18172-s1.doc]

**Magnetically controlled planar hyperbolic metamaterials for subwavlength resolution**

Bo Han Cheng, Hong Wen Chen, Kai Jiun Chang, Yung-Chiang Lan,

and Din Ping Tsai

**Appendix A:**

In region 1 (InSb) of Figure S1, the relative permittivity tensor can be written as,

$$\left[\varepsilon_{1}\right]=\left[\begin{matrix} \varepsilon_{xx} & \varepsilon_{xy} & 0 \\ -\varepsilon_{xy} & \varepsilon_{xx} & 0 \\ 0 & 0 & \varepsilon_{zz} \end{matrix}\right]$$

where$\varepsilon_{xx}=1-\frac{\omega_{p}^{2}}{\omega^{2}-\omega_{c}^{2}},\varepsilon_{xy}=\frac{-i\omega_{c}\omega_{p}^{2}}{\omega(\omega^{2}-\omega_{c}^{2})},$and $\varepsilon_{zz}=1-\frac{\omega_{p}^{2}}{\omega^{2}}.$The corresponding inverse matrix of $\left[\varepsilon_{1}\right]$ is

$\left[\varepsilon_{1}\right]^{-1}=\left[\begin{matrix} \frac{\varepsilon_{xx}}{\varepsilon_{xx}^{2}+\varepsilon_{xy}^{2}} & \frac{-\varepsilon_{xy}}{\varepsilon_{xx}^{2}+\varepsilon_{xy}^{2}} & 0 \\ \frac{\varepsilon_{xy}}{\varepsilon_{xx}^{2}+\varepsilon_{xy}^{2}} & \frac{\varepsilon_{xx}}{\varepsilon_{xx}^{2}+\varepsilon_{xy}^{2}} & 0 \\ 0 & 0 & \frac{1}{\varepsilon_{zz}} \end{matrix}\right]$.

In region 2 (dielectric), the relative permittivity is $\varepsilon_{2}.$ Assuming that the $H_{z}$ field is express as

$H_{z}=e^{i(k_{y}y-\omega t)}$, (A1)

and substituting Eq. (A1) into the wave equation listed below

$\nabla\times\left[\varepsilon\right]^{-1}\nabla\times H_{z}=(\frac{\omega}{c})^{2}H_{z}$, (A2)

then the following equation in region 1 is obtained

$\begin{matrix} \frac{-\partial}{\partial x}\left(\frac{\varepsilon_{xx}}{\varepsilon_{xx}^{2}+\varepsilon_{xy}^{2}}\right)\frac{\partial H_{z}}{\partial x}+\frac{\partial}{\partial x}\left(\frac{\varepsilon_{xy}}{\varepsilon_{xx}^{2}+\varepsilon_{xy}^{2}}\right)\frac{\partial H_{z}}{\partial y}-\frac{\partial}{\partial y}\left(\frac{\varepsilon_{xx}}{\varepsilon_{xx}^{2}+\varepsilon_{xy}^{2}}\right)\frac{\partial H_{z}}{\partial y} \\ -\frac{\partial}{\partial y}\left(\frac{\varepsilon_{xy}}{\varepsilon_{xx}^{2}+\varepsilon_{xy}^{2}}\right)\frac{\partial H_{z}}{\partial x}=(\frac{\omega}{c})^{2}H_{z} \end{matrix}$.

Reorganizing above equation yields

$\frac{d^{2}H_{z1}}{dx^{2}}+\gamma_{1}^{2} H_{z1}=0$ and $\gamma_{1}^{2} =(\frac{\omega}{c})^{2}\varepsilon_{\nu}-k_{y}^{2}$; where $\varepsilon_{\nu}=\frac{\varepsilon_{xx}^{2}+\varepsilon_{xy}^{2}}{\varepsilon_{xx}}$.

The corresponding general solution is

$H_{z1}=Ae^{i\gamma_{1}x}+Be^{-i\gamma_{1}x}$. (A3)

Similarly, in region 2, the following equation is also derived

$\frac{d^{2}H_{z2}}{dx^{2}}+\gamma_{2}^{2} H_{z2}=0$ and $\gamma_{2}^{2} =(\frac{\omega}{c})^{2}\varepsilon_{2}-k_{y}^{2}$.

And the corresponding general solution is

$H_{z2}=Ce^{i\gamma_{2}x}+De^{-i\gamma_{2}x}$. (A4)

Next, considering the Maxwell–Faraday equation $\nabla\times H=-i\omega\left[\varepsilon\right]E$, the y components of electric field in the region 1 and 2 are also obtained, respectively, as

$E_{y1}=\frac{-1}{i\omega(\varepsilon_{xx}^{2}+\varepsilon_{xy}^{2})}\left[A(\varepsilon_{xy}k_{y}-\varepsilon_{xx}\gamma_{1})e^{i\gamma_{1}x}+B(\varepsilon_{xy}k_{y}+\varepsilon_{xx}\gamma_{2})e^{-i\gamma_{1}x}\right]$ (A5)

$E_{y2}=\frac{\gamma_{2}}{\omega\varepsilon_{2}}(Ce^{i\gamma_{2}x}-De^{-i\gamma_{2}x})$ (A6)

Applying the boundary conditions of electric and magnetic fields, that is,

$\begin{matrix} H_{z1}(d_{1})=H_{z2}(d_{1}) \\ E_{y1}(d_{1})=E_{y2}(d_{1}) \\ H_{z1}(0)=H_{z2}(a)e^{-ik_{x}a} \\ E_{y1}(0)=E_{y2}(a)e^{-ik_{x}a} \end{matrix}$,

to Eq. (A3), (A4), (A5), and (A6), then the requirement of nontrivial solution results in the following equation

$\left|\begin{matrix} e^{i\gamma_{1}d_{1}} & e^{-i\gamma_{1}d_{1}} & -e^{i\gamma_{2}d_{1}} & -e^{-i\gamma_{2}d_{1}} \\ 1 & 1 & -e^{i(\gamma_{2}-k_{x})a} & -e^{-i(\gamma_{2}+k_{x})a} \\ \frac{-i\gamma_{1}e^{i\gamma_{1}d_{1}}}{\varepsilon_{1}} & \frac{i\gamma_{1}e^{-i\gamma_{1}d_{1}}}{\varepsilon_{1}} & \frac{-i(\varepsilon_{xy}k_{y}-\gamma_{2}\varepsilon_{xx})e^{i\gamma_{2}d_{1}}}{\varepsilon_{xx}^{2}+\varepsilon_{xy}^{2}} & \frac{-i(\varepsilon_{xy}k_{y}+\gamma_{2}\varepsilon_{xx})e^{-i\gamma_{2}d_{1}}}{\varepsilon_{xx}^{2}+\varepsilon_{xy}^{2}} \\ \frac{-i\gamma_{1}}{\varepsilon_{1}} & \frac{i\gamma_{1}}{\varepsilon_{1}} & \frac{-i(\varepsilon_{xy}k_{y}-\gamma_{2}\varepsilon_{xx})e^{i(\gamma_{2}-k_{x})a}}{\varepsilon_{xx}^{2}+\varepsilon_{xy}^{2}} & \frac{-i(\varepsilon_{xy}k_{y}+\gamma_{2}\varepsilon_{xx})e^{-i(\gamma_{2}+k_{x})a}}{\varepsilon_{xx}^{2}+\varepsilon_{xy}^{2}} \end{matrix}\right|=0.$ (A7)

Reorganizing Eq. (A7) finally yields the isofrequency dispersion relation

$\cos(k_{x}a)=\cos(d_{1}\gamma_{1})\cos(d_{2}\gamma_{2})-\frac{1}{2}(\frac{-k_{y}^{2}\varepsilon_{1}\varepsilon_{xy}}{\gamma_{1}\gamma_{2}\varepsilon_{2}\varepsilon_{xx}}+\frac{\gamma_{2}\varepsilon_{1}}{\gamma_{1}\varepsilon_{2}}+\frac{\gamma_{1}\varepsilon_{2}}{\gamma_{2}\varepsilon_{1}})\sin(d_{1}\gamma_{1})\sin(d_{2}\gamma_{2}).$ (A8)


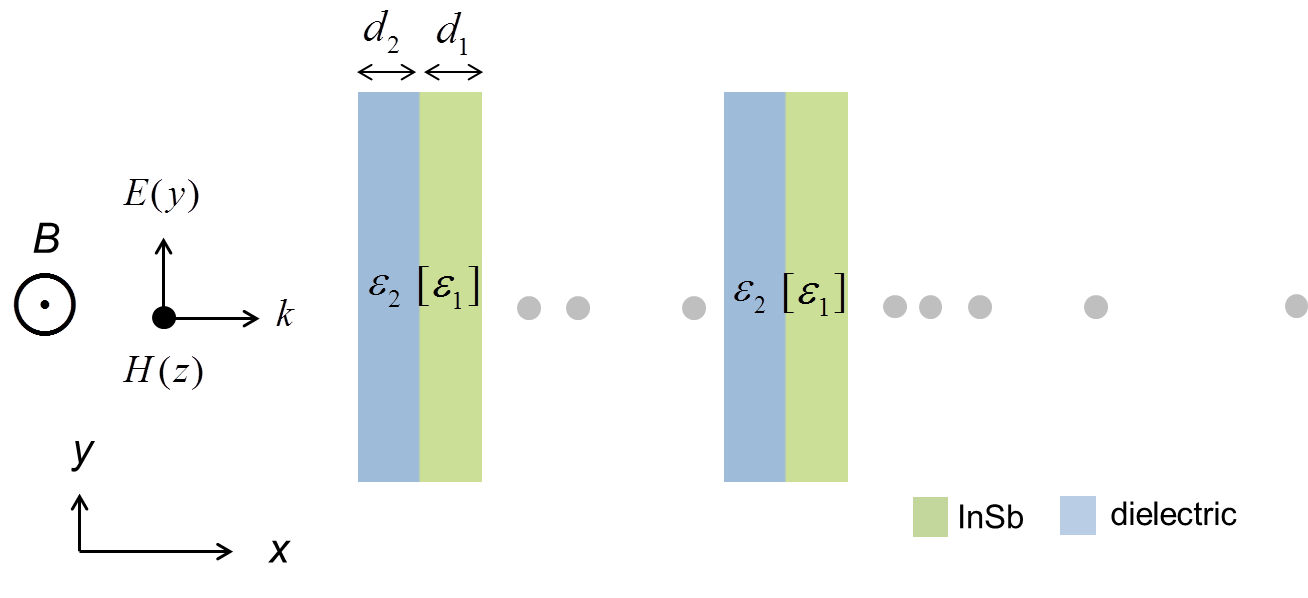


**Figure S1.** Schematic InSb-dielectric multi-layered structure under Voigt configuration.

**Appendix B:**


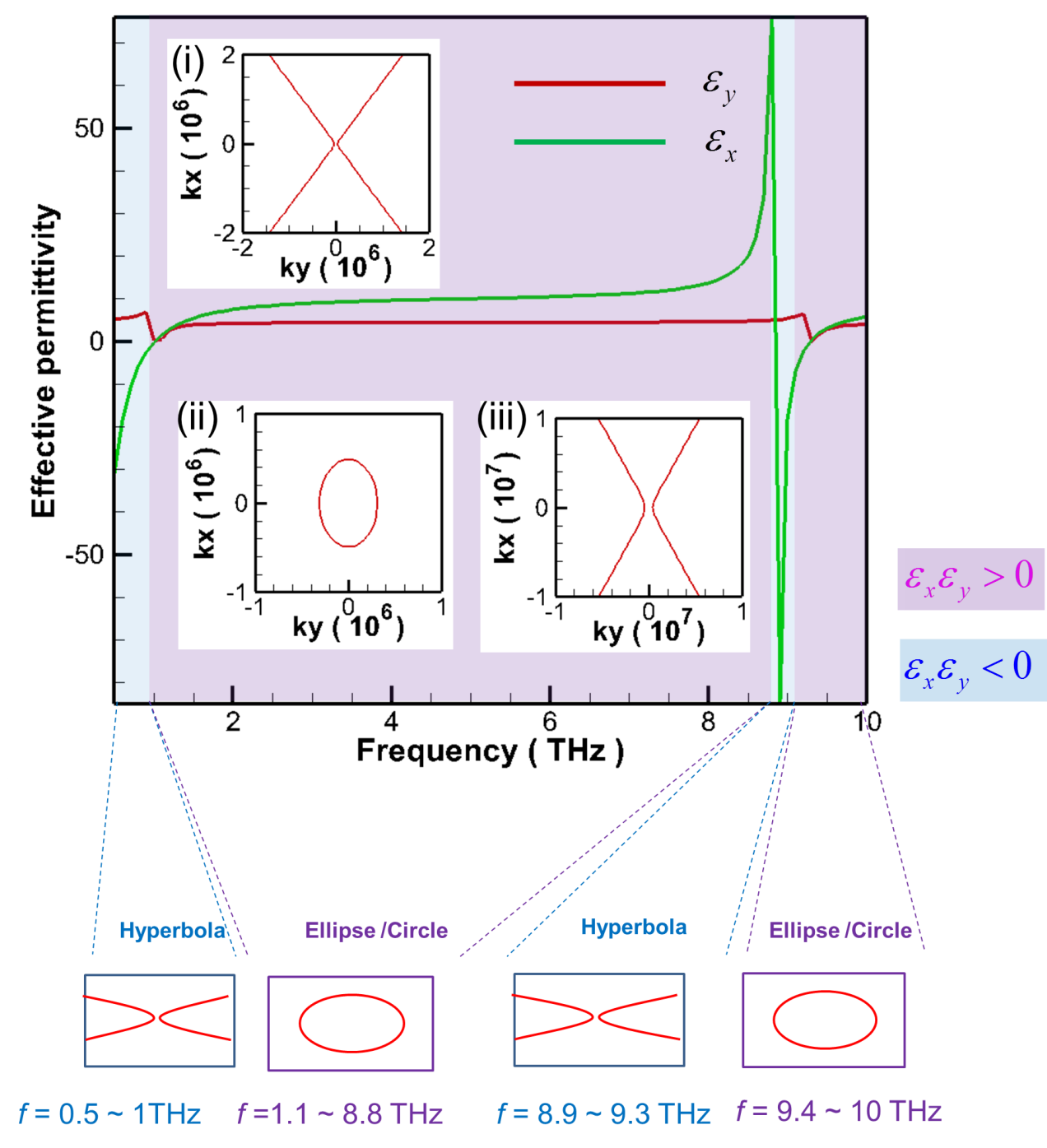


**Figure S2.** Real parts of effective permittivities ($\varepsilon_{x}$ and $\varepsilon_{y}$) as a function of incident frequency in the layered structure at 4.14 Tesla. Insets (i), (ii), and (iii): Calculated isofrequency dispersion curves at 0.7, 7, and 9 THz, respectively. The corresponding real parts of ($\varepsilon_{x}$, $\varepsilon_{y}$) are (5.56, -10.95), (4.44, 11.22), and (5.33, -18.4176). The isofrequency dispersion curve is hyperbolic when the condition $\varepsilon_{x} \varepsilon_{y}<0$ is fulfilled. Conversely, as $\varepsilon_{x} \varepsilon_{y}>0$, the dispersion curve becomes elliptic.

**Appendix C:**

Figures S3 and S4 plot the calculated dispersion relations ($\omega-k$ diagrams) of SPs and SMPs at the InSb-PMMA interface for the case of Fig. 3(f) (the relative permittivity of PMMA is 2.5). Black (red and green) solid line presents the dispersion relation under the magnitude of magnetic field *B* equal to 0 Tesla (4.14 Tesla). For *B* = 0 Tesla, the dispersion curve is symmetrical to *k* = 0 axis. The SPs that are excited by a TM-polarized plane wave incident on the Cr film can simultaneously propagate along +y and –y directions as show in insets (ii) and (iv) of Figs. S3 and S4. However, for *B* = 4.14 Tesla, the dispersion curve becomes asymmetric. When the applied magnetic field is along –z direction (Fig. S3), the cut-off frequency of left-propagation (right-propagation) mode increases from 2.94 THz to 9.13 THz (decreases from 2.94 to 0.95 THz). Therefore, for incident frequencies of 1 THz and 7.95 THz, only the left-propagation mode can exist. They are also verified by numerical simulations as shown in inset (i) and (iii) of Fig. S3. When the direction of the applied magnetic field changes from -*z* to *z*, the propagation property of SMPs will be reversed (i.e. only the right-propagation mode exists as shown in Fig. S4).


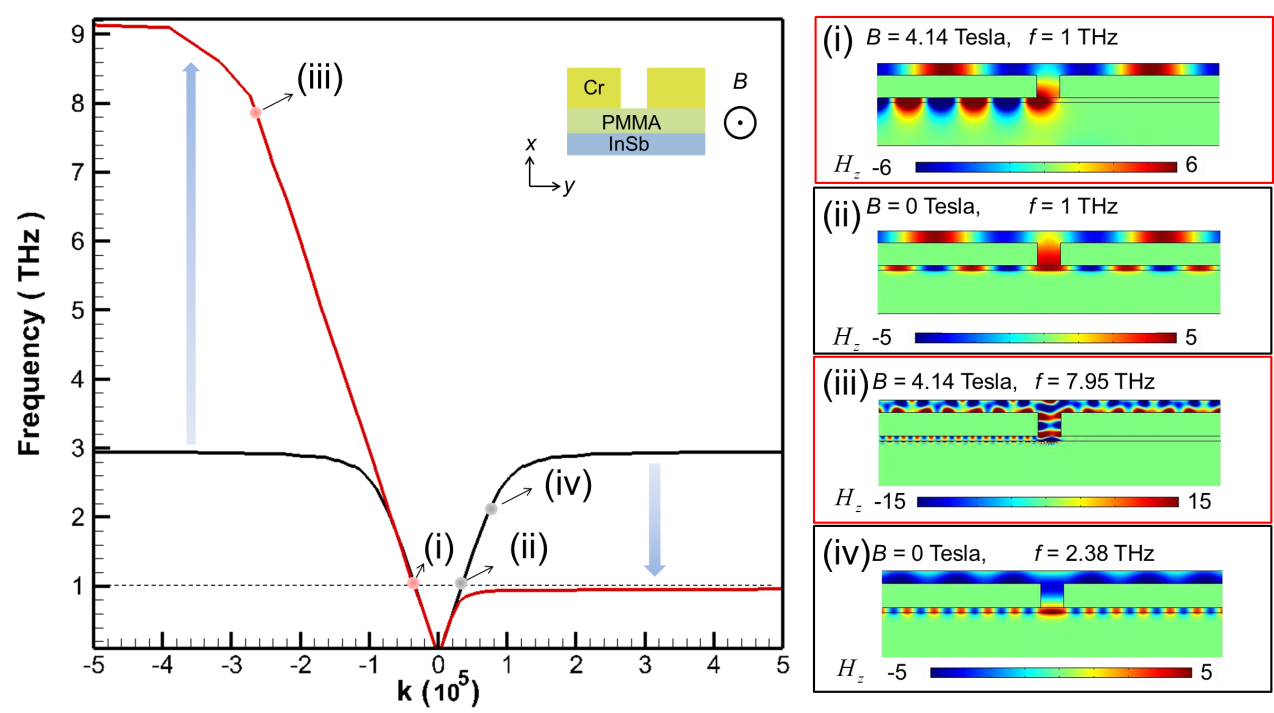


**Figure S3.** Calculated dispersion relations ($\omega-k$ diagrams) for SPs (*B* = 0 Tesla, solid black line) and SMPs (*B* = 4.14 Tesla, solid red line) at InSB-PMMA interface. The applied magnetic field is along -*z*-direction. Black dashed line denotes the operation frequency of 1 THz. Insets (i) – (iv): Simulated *Hz* field contours at various values of magnetic field and frequency.


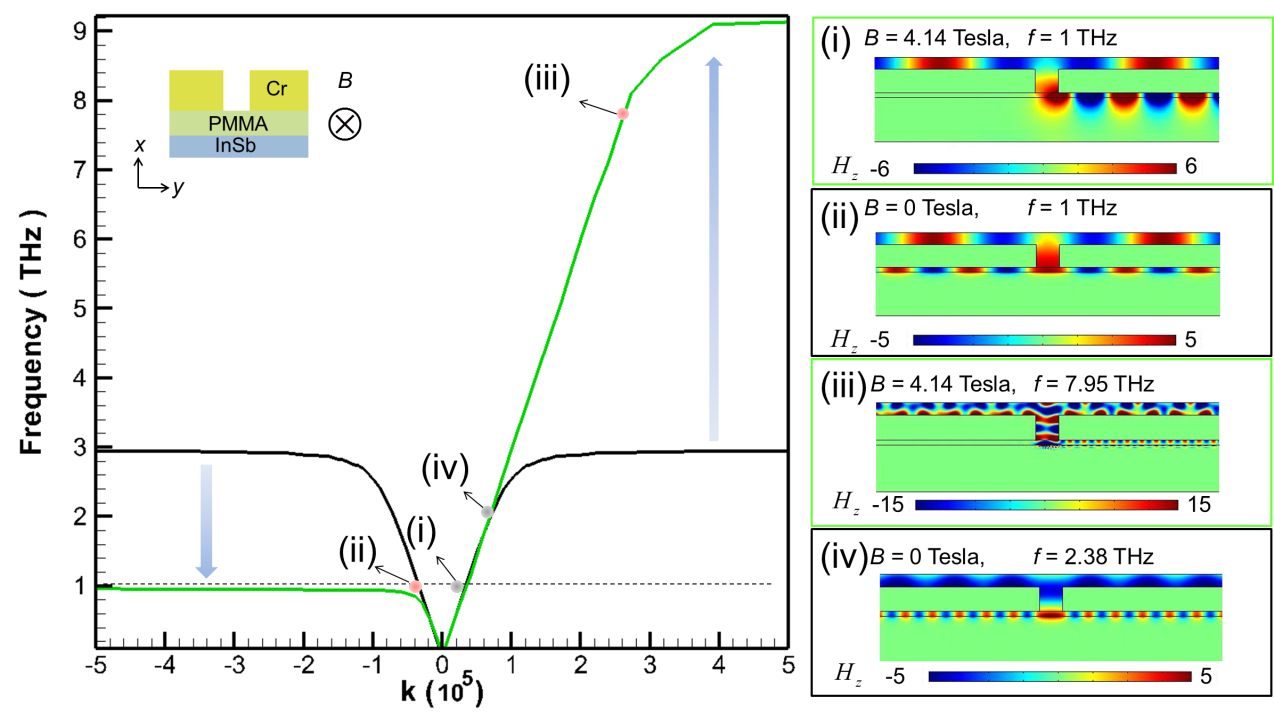


**Figure S4.** Calculated dispersion relations ($\omega-k$ diagrams) for SPs (*B* = 0 Tesla, solid black line) and SMPs (*B* = 4.14 Tesla, solid green line) at InSB-PMMA interface. The applied magnetic field is along +*z*-direction. Black dashed line denotes the operation frequency of 1 THz. Insets (i) – (iv): Simulated *Hz* field contours at various values of magnetic field and frequency.

**Appendix D:**

Figures S5(a) – S5(d) present the simulated normalized intensities versus y position measured at the dashed yellow lines in Figs. 6 for the double slits embedded in air, embedded in Si, on Si plate and on the proposed layered structure, respectively. Figure S5 indicates that the double slits only can be distinguished by the proposed layered structure with *B* = 4.14 Tesla at this x position.


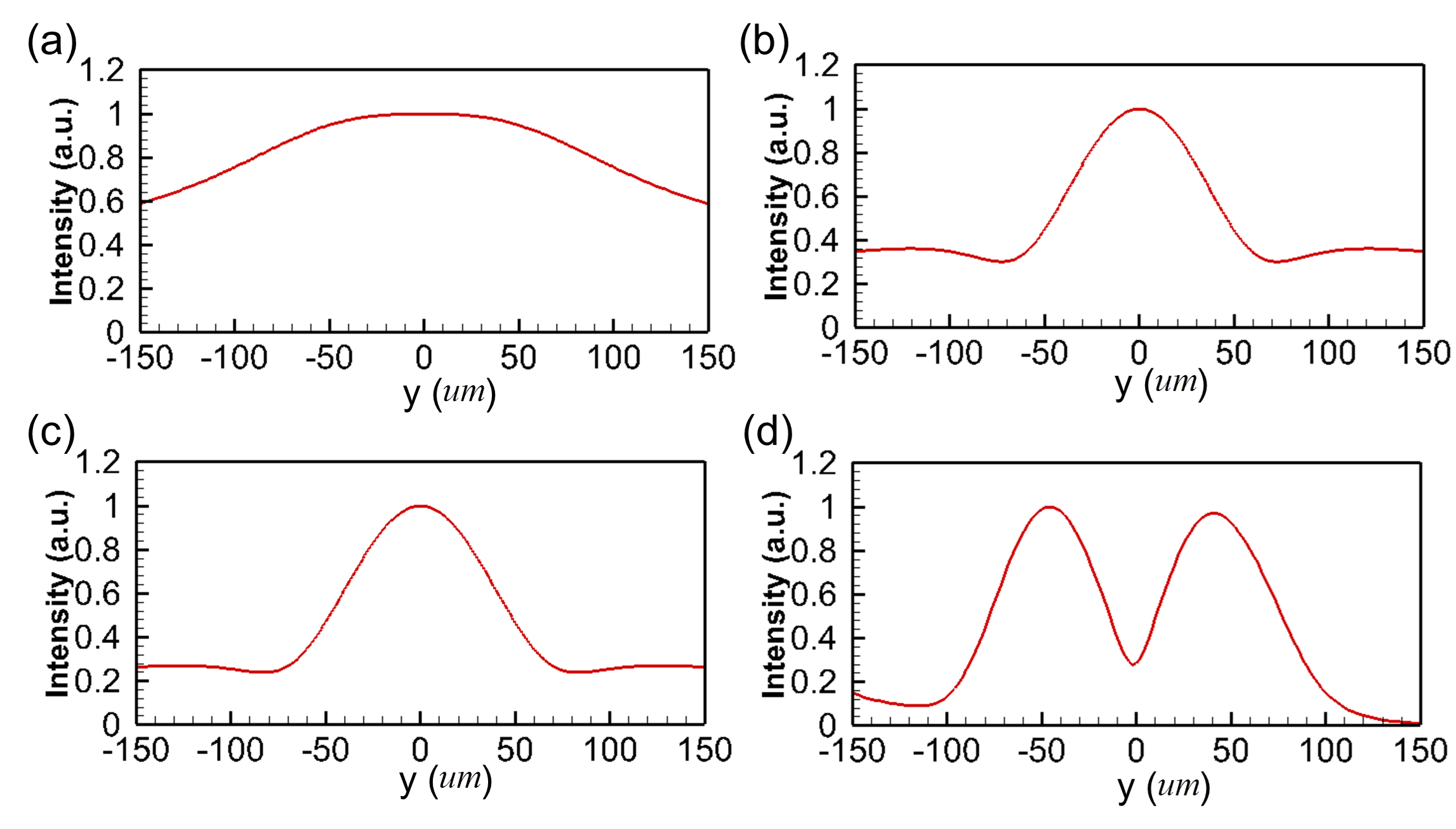


**Figure S5.** Simulated normalized intensities versus y position measured at the dashed yellow lines in Figs. 6 for double slits embedded in different materials at incident frequency of 1 THz. (a) and (b) The double slits are embedded in air and in Si, respectively. (c) and (d) The double slits are on Si plate and on proposed layered structure with *B* = 4.14 Tesla, respectively. *L* = 80 $\mu$m.

**Appendix E:**

Figure S6 plots the simulated intensity of propagating wave in the layered structure as a function of x position with *L* = 80 $\mu$m and *B* = 4.14 Tesla at incident frequency of 1 THz. The measured y position is at the center of the left slit (the dashed white line in inset of Fig. S6). Figure S6 reveals that the intensity of the propagating wave at the x position of image measurement in Fig. S5 is reduced to about one-twentieth of that at the exit of slit.


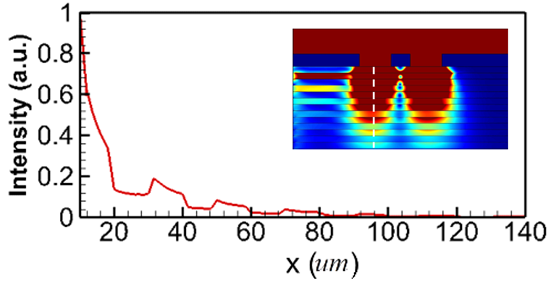


**Figure S6.** Simulated intensity of propagating wave in the layered structure as a function of x position with *L* = 80 $\mu$m and *B* = 4.14 Tesla at 1 THz. The origin of x axis is at the exit of slit. Inset: The measured y position is at the center of the left slit (the dashed white line).
